# Chitosan nanoparticles containing limonene and limonene-rich essential oils: potential phytotherapy agents for the treatment of melanoma and breast cancers

**DOI:** 10.1186/s12906-021-03362-7

**Published:** 2021-07-02

**Authors:** Hiva Alipanah, Mojtaba Farjam, Elham Zarenezhad, Ghazaal Roozitalab, Mahmoud Osanloo

**Affiliations:** 1 Department of Physiology, School of Medicine, Fasa University of Medical Sciences, Fasa, Iran; 2 Noncommunicable Diseases Research Center, Fasa University of Medical Sciences, Fasa, Iran; 3 Clinical Research Development Unit, Valie-Asr Hospital, Fasa University of Medical Sciences, Fasa, Iran; 4 Department of Medical Nanotechnology, School of Advanced Technologies in Medicine, Fasa University of Medical Sciences, Fasa, Iran

**Keywords:** *Citrus*, Triple negative breast neoplasms, Skin neoplasms, Phytochemicals, Cytostatic agents

## Abstract

**Background:**

Melanoma and breast cancers are two common cancers worldwide. Due to the side effects of chemotherapy drugs and the occurring resistance against them, the development of green drugs has been received more attention.

**Methods:**

The anticancer effects of three essential oils from the *Citrus* family and their identified major constituents (limonene) were first investigated against melanoma and breast cancer cell lines (A-375 and MDA-MB-468). By preparing chitosan nanoparticles containing them, an attempt was then made to improve their effectiveness.

**Results:**

Chitosan nanoparticles containing *Citrus sinensis* and *Citrus limon* essential oils with IC_50_s of 0.03 and 0.124 μg/mL on A-375 cells, and 23.65 and 40.32 μg/mL on MDA-MB-468 showed distinct anticancer efficacies.

**Conclusion:**

The prepared formulations could thus be considered as green anticancer agents in complementary medicine and therapies.

## Background

Breast cancer is one of the most common cancer, followed by prostate, lung, and colon cancers worldwide [1]. Breast cancer is the commonest life-threatening malignancy, causing 14% of all cancer-related fatalities among women [2, 3]. Skin cancer is another common and preventable carcinoma worldwide; its annually rising incidence has made it a pre-eminent public health threat [4]. Malignant melanoma is a type of skin cancer and responsible for the vast majority of skin cancer deaths; it begins with the abnormal proliferation of cells known as melanocytes [5]. Chemotherapy, surgery, and radiotherapy are common cancer treatments, but their nonspecific action and severe side effects are cancer treatment’s biggest obstacles [6, 7]. To bypass the side effects, developing new green drugs, especially essential oils (EO) s, has recently received more attention [8, 9].

*Citrus aurantium* (bitter orange) is a tree 4–6 m high, evergreen, hairless, with long blades of the *Rutaceae* (*Citrus*) family [10]. It possesses antispasmodic, anti-inflammatory, anti-flatulence, antihypertensive, and diuretic properties [11–13]. *Citrus limon* (L.) or lemon is another member of the *Rutaceae* family rich in vitamins, minerals, dietary fiber, and carotenoids [14]. *Citrus sinensis* (scientifically name for the orange) grows as a fruit-bearing shrub with green leaves and white flowers and is also a member of the *Rutaceae* family [15, 16]. EOs of the three mentioned specimens possesses anticancer effects against different types of breast cancer cell lines. Their IC_50_s against MCF-7 were reported at 82, 57, and 39 μg/mL, their IC_50_s against MDA-MB-231 were reported at 74, 37, and 39 μg/mL, and their IC_50_s against T47D were reported at > 300, 19, and 43 μg/mL [17]. Moreover, limonene (1-methyl-4-(1-methylethenyl) cyclohexane) is a major constituent in the mentioned EOs; it is a colorless and aromatic liquid oil and acts as a potential chemotherapeutic monocyclic monoterpene in nature [18–20]. Limonene has significant anticancer activity by inhibiting tumor initiation, growth and angiogenesis, and cancer cell apoptosis [21, 22]. For instance, the antiproliferative activity of limonene on BW5147 cells, colon, gastro, melanoma, mammary gland tumors has been confirmed [23, 24].

In this research, the anticancer effects of *C. aurantium, C. limon,* and *C. sinensis* EOs (CAEO, CLEO, and CSEO) and limonene were first investigated against A-375 (human melanoma cancer cell line) and MDA-MB-468 (human breast cancer cell line). By preparing chitosan nanoparticles (ChiNPs) containing them, an attempt was made to improve their anticancer properties.

## Methods

### Cells and reagents

Pasteur Institute of Iran supplied the used cell lines, including breast cancer cell line MDA-MB-468 (ATCC HTB-132) and melanoma cell line A-375 (ATCC CRL-1619). Tetrazolium salt, 3-(4,5-dimethyl-thiazol-2-yl)-2,5-diphenyltetrazo-lium bromide (MTT), phosphate-buffered saline (PBS) tablets, Sodium-tripolyphosphate (TPP), chitosan low molecular weight, acetic acid, tween 20, and limonene were purchased from Sigma-Aldrich (USA). Penicillin streptomycin, trypsin, dimethyl sulfoxide (DMSO), and Dulbecco’s Modified Eagle’s Media (DMEM) cell culture medium were purchased from Shellmax (China). Fetal bovine serum (FBS) was obtained from Gibco (USA). EOs were purchased from Iranian companies; CSEO Green Plants of Life Co. Ltd., CLEO Barij Essence Pharmaceutical Co., and CAEO Tabib Daru Co.

### Chemical composition of the EOs

Ingredients of CSEO, CLEO, CAEO were analyzed by *Gas Chromatography-Mass Spectrometry* (*GC–MS*)*;* the EOs were analyzed using a 6890 GC system coupled with a 5975 series of *mass selective detectors* (Agilent Technologies, USA). The separations were performed on HP-5MS silica fused column (length, 30 m; internal diameter, 0.25 mm; film thicknesses, 0.25 mm; stationary phase, 5% phenyl 95% methyl polysiloxane). The column temperature program started at 40 °C (fixed for 1 min), then raised with a rate of 3 °C min^− 1^ to 250 °C, and was finally held for 60 min at this temperature. The temperature of the injection port and detector was fixed at 250 and 230 °C, respectively. Other operating conditions were as follows: carrier gas, helium (99.999%), split-flow 25 mL/min, septum purge 6 mL/min, and column flow rate 1 mL/min. Mass spectra were taken at full scan mode and 70 eV ionization energy and full scan mode. The scanned mass range was set at 50–550 m/z. The identification of ingredients was performed as described in our previous study, and the peak area normalization procedure was used for the quantitative determination of the compounds in the EOs [25].

### Preparation of chitosan nanoparticles

**Fig. 1:**
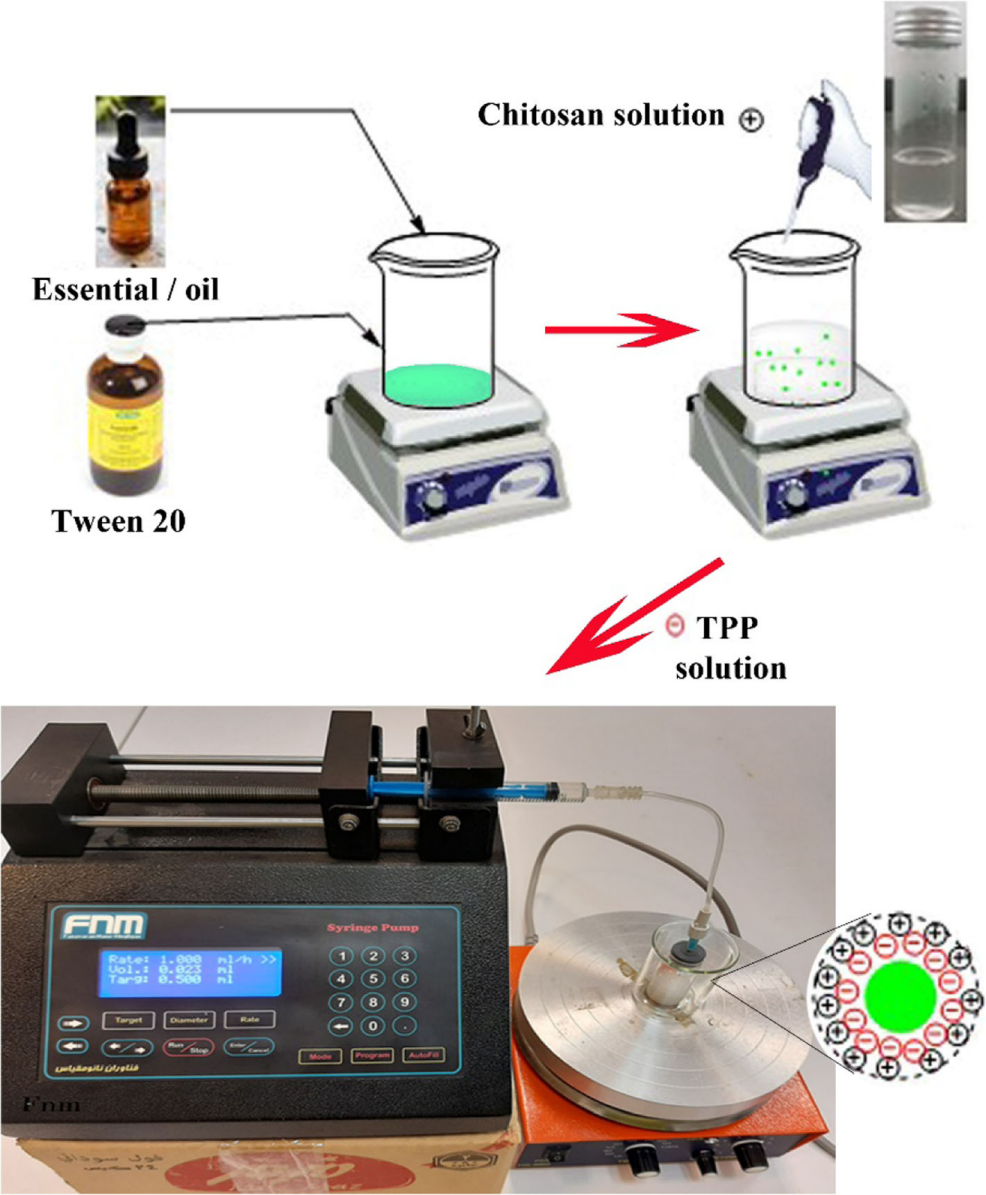
Steps of preparation of chitosan nanoparticles containing EOs or limonene

Chitosan powder (0.25% w/v) was dissolved in a 1% acetic acid aqueous solution (4 h, 2000 rpm, ambient temperature). Preparation of chitosan nanoparticles containing limonene, CAEO, CLEO, and CSEO was obtained using a modified ionic gelation technique, as depicted in Fig. 1 [26]. Each EO or limonene (0.5% w/v) and tween 20 (0.5% w/v) was first mixed at 2000 *rpm* for 30 min at room temperature. The chitosan solution was then added and stirred for another 30 min. After that, an aqueous solution of TPP (0.15% w/v) was added 1 mL/h using the syringe pump, and the mixture was stirred for 40 min (2000 rpm) to stabilizing the nanoparticles. The prepared samples were abbreviated as CLChiNPs, CSChiNPs, CAChiNPs, and LimChiNPs. Moreover, the same methodology was used to prepare free chitosan nanoparticles (ChiNPs), but no EO or limonene was used.

### Characterization of the prepared nanoformulation

#### Size analysis

Dynamic light scattering (DLS) technique was used to analyze all nanoformulations particle sizes (dynamic light scattering, scatter scope, K-ONE NANO. LTD, Korea). D50 was considered particle size, and particle size distribution (SPAN) was calculated as d90-d10/d50. D is diameter, and 10, 50, and 90 are percentile of particles with a smaller diameter than these specified diameters. Formulations with SPAN values less than 1 possess narrow particle size distributions [27].

#### The attenuated Total reflection-Fourier transform infrared (ATR-FTIR) analysis

ATR-FTIR has analyzed the loading of the EOs or limonene in the ChiNPs. Spectra of ChiNPs, limonene, limChiNPs, CSEO, CSChiNPs, CLEO, CLChiNPs, CAEO, and CAChiNPs were recorded in 400–4000 cm^− 1^. The samples without preparation were subjected to the instrument (FTIR, Bruker Company, Model Tensor II, Germany).

### Investigation of the anticancer activity

The anticancer activity of limonene, CAEO, CLEO, and CSEO (as non-formulated samples) also, LimChiNPs, CAChiNPs, CLChiNPs, and CSChiNPs (as nanoformulations) were investigated using MTT assay. The EOs and limonene were dissolved (0.5% w/v) in a PBS solution containing 0.5% DMSO. The cell lines were cultured in 75 cm^2^ culture flasks in DMEM medium supplemented with 10% of FBS and 1% of penicillin-streptomycin (P/S) and incubated at 37 °C air (95%) and CO_2_ (5%). Cells (MDA-MB-468 and A-375) were separated by trypsin; they were then seeded (1 × 10^4^ cells per well) in 96 well plates and incubated overnight for attachment. The following day, the culture media was discarded, and a 75 μL complete fresh medium was added to each well. By adding an appropriate amount of the samples, concentrations were finally fixed at 1200, 600, 300, 150, and 75 μg/mL. The treated plates were incubated for 24 h at 37 °C with CO_2_ 5%, and their content was then discarded, and wells were washed with 100 μL PBS to remove nanoformulations milky color. After that, 100 μL MTT reagent (0.5 mg.mL^− 1^) was added to each well and were incubated for another 4 h; created formazan crystals were then dissolved by adding DMSO (100 μL/well). The control group (six-well in each plate) was treated only with 25 μL PBS (0.5% DMSO) and 75 μL culture media. Finally, the absorbance (A) of the wells was monitored by an ELISA plate reader at 570 nm. The cell viability at each concentration was calculated using the following equation:

1 $$ \mathrm{Cell}\ \mathrm{viability}\ \left(\%\right)=\frac{Mean\ A\ s\mathrm{a} mple}{Mean\ A\  control}\times 100 $$

## Results

### Identified compounds in used EOs

Limonene was identified as the major compound in all three used EOs; it comprised 31.4% of CAEO, 61.8% of CLEO, and 71.3% of CSEO (see Table 1). Sabinene (15.6%), ɣ-terpinene (6.0%), linalool (5.6%), and nerolidol (5.1%) were the other four major compounds of CAEO. Alpha-pinene, sabinene, *cis*-limonene oxide, and *trans*-limonene oxide with a portion of 3.5, 17.0, 2.3, and 3.1% were the other four CLEO compounds. In SCEO, *trans*-p-2,8-Menthadien-1-ol, *cis*-limonene oxide, *trans*-limonene oxide, and *trans*-carveol were identified as the other four major compounds (5.0, 2.6, 2.3, and 2.9%).

**Table 1:** Identified compounds with more than 1% in EOs of *C. aurantium, C. limon*, and *C. sinensis* (CAEO, CLEO, and CSEO)

RT^**a**^	RI^**b**^	Compound	CAEO		CLEO		CSEO	
			Area	%	Area	%	Area	%
9.4	622.7	α-pinene	58,484,827	1.7	126,834,642	3.5	37,874,128	1.2
11.3	694.5	sabinene	542,668,432	15.6	623,674,861	17.0	34,246,642	1.1
11.3	696.9	β-pinene	43,301,938	1.2	–	–	–	–
12.0	714.9	β-myrcene	108,784,770	3.1	–	–	–	–
13.1	742.0	α-terpinene	59,177,141	1.7	–	–	–	–
13.9	762.4	limonene	1,088,445,097	31.4	2,269,351,083	61.8	2,266,978,799	71.3
14.7	781.9	β-ocimene Y	162,728,160	4.7	–	–	–	–
15.1	793.5	ɣ-terpinene	207,125,216	6.0	–	–	–	–
17.2	835.8	linalool	192,637,034	5.6	–	–	–	–
18.1	853.7	*cis*-p-Menth-2,8-dienol	–	–	–	–	60,702,174	1.9
18.6	864.7	*trans*-p-2,8-Menthadien-1-ol	–	–	–	–	157,669,843	5.0
18.8	868.0	*cis*-limonene oxide	–	–	83,422,623	2.3	82,287,580	2.6
18.8	869.1	*trans*-limonene oxide	–	–	113,204,736	3.1	72,968,453	2.3
20.6	905.3	4-terpineol	66,692,764	1.9	–	–	–	–
21.5	921.1	nortricyclene	–	–	–	–	31,832,189	1.0
22.7	943.8	*trans*-carveol	–	–	–	–	92,456,195	2.9
23.2	953.7	*cis*-carveol	–	–	–	–	55,583,810	1.7
23.6	960.1	cumaldehyde	128,003,231	3.7	–	–	–	–
23.7	963.5	carvone	–	–	–	–	66,472,215	2.1
25.8	1002.3	3-buten-1-ol, 4-chloro-2-methyl-1-phenyl	41,752,965	1.2	–	–	–	–
28.2	1047.1	1,2-yclohexanediol, 1-methyl-4-(1methylethenyl)	–	–	55,304,649	1.5	–	–
37.1	1223.5	nerolidol	178,234,586	5.1	–	–	–	–
42.9	1345.6	farnesol	34,776,356	1.0	–	–	–	–

^a^retention time, ^b^retention index

### The particle size of the prepared chitosan nanoparticles containing limonene or EOs

**Fig. 2:**
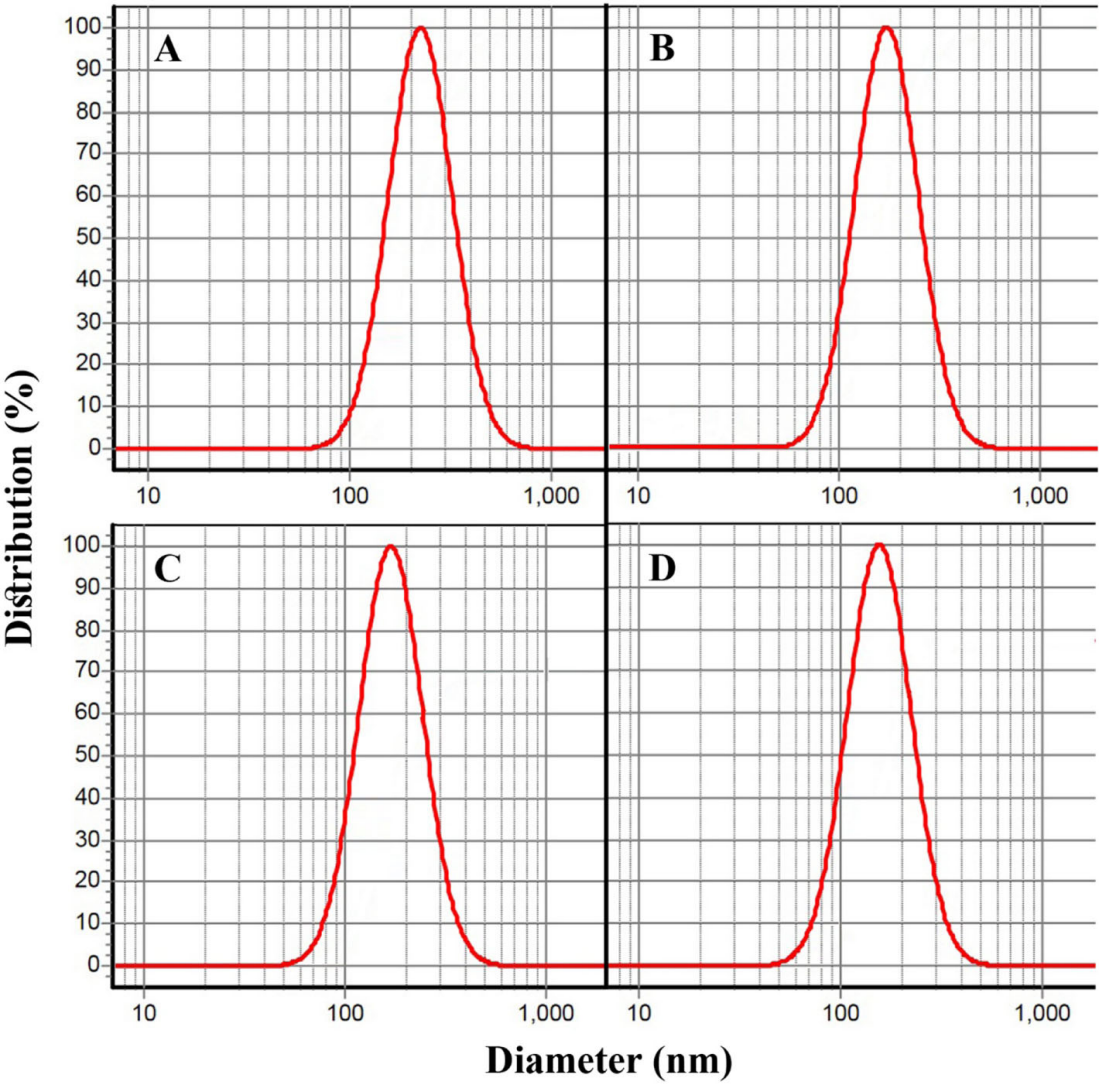
Chitosan nanoparticles containing limonene and EOs of *C. aurantium, C. limon*, and *C. sinensis*: **A**) LimChiNPs 209 ± 13 nm, **B**) CAChiNPs 173 ± 6 nm, **C**) CLChiNPs 181 ± 4 nm, **D**) CSChiNPs 156 ± 8 nm

DLS analyses of the prepared nanoformulations are given in Fig. 2. CSChiNPs with a particle size of 156 ± 8 nm (SPAN 0.92) possess the smallest particles. CAChiNPs (173 ± 6 nm), CLChiNPs (181 ± 4 nm), and LimChiNPs (209 ± 13 nm) were situated in other ranks; their SPAN values were including 0.94, 0.90, 0.93, respectively.

### Successful loading of limonene or EOs in chitosan nanoparticles

**Fig. 3:**
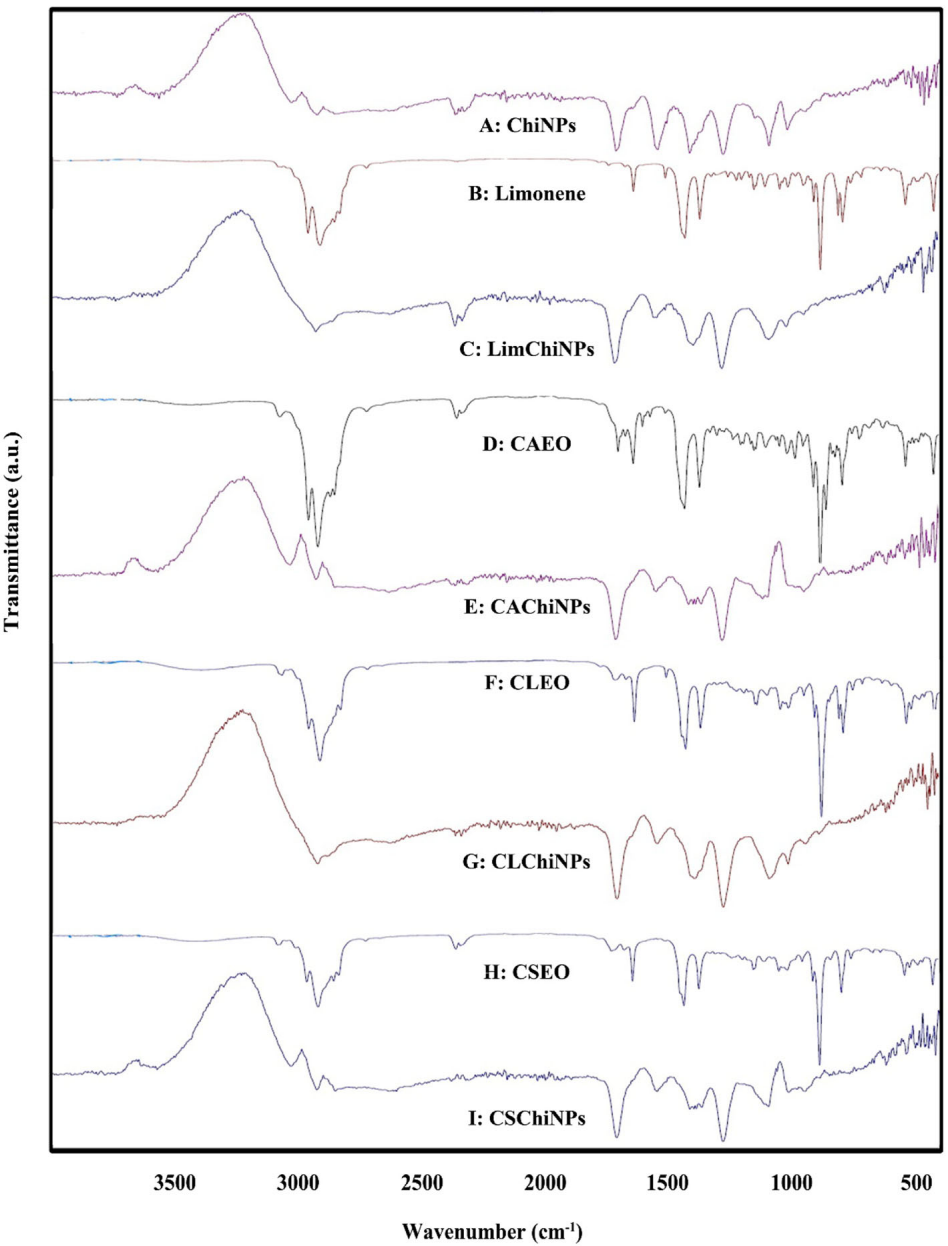
ATR-FTIR of samples; **A**) chitosan nanoparticles, **B**) limonene, **C**) ChiNPs containing limonene, **D**) *C. aurantium* EO, **E**) ChiNPs *C. aurantium* EO, **F**) *C. limon* EO, **G**) ChiNPs containing *C. limon* EO, **H**) *C. sinensis* EO, and **I**) ChiNPs containing *C. sinensis* EO

The ATR-FTIR spectrum of ChiNPs is depicted in Fig. 3A; the bond at about 1700 cm-1 can be related to carbonyl stretching of the pure chitosan’s secondary amide band and carbonyl group tween. The characteristic peak at 1094 cm^− 1^ relates to symmetric and anti-symmetric stretching vibrations in the PO_2_ group, and the strong band at 1020 cm^− 1^ belongs to symmetric and anti-symmetric stretching vibrations in the PO_3_ group. After the crosslinking process, two bands at 1280 and 1152 cm^− 1^ belong to anti-symmetric stretching vibrations of PO_2_ groups in TPP ions. These new peaks confirmed ionic crosslinks between protonated amino groups of chitosan and tripolyphosphate anionic groups.

In the ATR-FTIR spectrum of limonene (Fig. 3B), the band at 3001 cm^− 1^ relates to = C-H, and the bands at 2962, 2913, 2855, and 2855 cm^− 1^ showed –CH stretching, the specific absorption at 1677 cm^− 1^ corresponds to the stretching vibration of vinyl substituted C=C. The absorption band at 1644 cm^− 1^ is assigned to the C=C vibration. The characteristic peaks at 989 and 890 cm^− 1^ attributed to C-H bending absorption.

In the spectrum of LimChiNPs (Fig. 3C), the characteristic peak at 2925 m^− 1^ is showed C-H stretching in chitosan and C-H of limonene. A strong characteristic peak showed at 1278 cm^− 1^ belong to C-N stretching indicates the complex formation via electrostatic interaction between NH3^+^ groups of chitosan within phosphoric groups of TPP. The band around 1580 cm^− 1^ can be assigned to C-N stretching vibration and refers to the amide group because of the NH_2_ bending vibration. The band at 1017 cm^− 1^ related to C-O in chitosan and the shape and position of the peaks proved that limonene was successfully encapsulated ChiNPs.

ATR-FTIR spectrum of CAEO is depicted in Fig. 3D; the broad peak at 3436 cm^− 1^ attributes to NH, the bands at 3761 related to = C-H. The bands at 2961, 2923, 2872 cm^− 1^ displayed –CH cm^− 1^, and the peak at 1706 cm^− 1^ corresponding to the stretching vibration of carbonyl C=O. Absorption bands at 1644 and 1437 cm-^1^ are assigned to the aromatic ring C=C skeleton vibration of an aromatic substance. The peak at 1022 cm^− 1^ is characteristic of a stretching vibration of C-N. The peak at 957 cm^− 1^ is attributed to C-H bending absorption, and the strong peak at 758 cm^− 1^ is assigned to benzene rings C-H vibration absorption.

From Fig. 3E, some of the specific peaks of CAEO disappeared when it was encapsulated in chitosan, i.e., CAChiNPs. A strong new peak showed at 1281 cm^− 1^ belong to the C-N stretch indicating the complex formation via electrostatic interaction between NH3^+^ groups of chitosan within phosphoric groups of TPP. Also, the band around 1547 cm^− 1^ can be assigned to C-N stretching vibration and refers to the amide group because of the NH_2_ bending vibration.

In ATR-FTIR spectra of CLEO, Fig. 3F, the bands at 3399 cm^− 1^ related to OH stretching vibration, the bands at 3072 cm^− 1^ related to = C-H. The bands at 2963, 2918, 2855, and 2834 cm^− 1^ showed –CH stretching vibration, and the characteristic bands 1677 cm^− 1^ corresponding to C=C, the peak at around 1105 cm^− 1^ related to C-O bending vibration.

In ATR-FTIR of CLChiNPs (Fig. 3G), the new strong peak at around 1280 cm^− 1^ relates to C-N stretching was attributed to the electrostatic interaction between chitosan and TPP. Also, the sharp peak around 1545 cm^− 1^ attributed to C-N stretching vibration related to the amide group because of the NH_2_ bending vibration. All other characteristic peaks appear in the spectra of CLEO and ChiNPs; it is confirmed that CLEO was successfully encapsulated in ChNPs.

In the spectrum of CSEO (Fig. 3H), the bond at about 3412 cm^− 1^ can be related to OH stretching vibrations, and the peaks at around 2964, 2918, 2885 cm^− 1^ are related to -C-H. The vibrational bands around 1729 cm^− 1^ related to C=O, and the band around 1677 attribute C-O stretching vibrations. The main peaks around 1110, 1115, and 1309 cm^− 1^ are related to C-O. The peaks at 885 cm^− 1^ attributed to C-H bending absorption.

After CSEO was encapsulated, the spectrum showed a strong peak at 1280 cm^− 1^ belongs to the C-N stretch, indicating the complex formation via electrostatic interaction between chitosan and TPP (see Fig. 3I). The strong band around 1547 cm^− 1^ can be assigned to C-N stretching vibration and refers to the amide group because of the NH_2_ bending vibration. The band at 1096 cm^− 1^ due to the phosphoric acid root and the protonation of amino cross-linking effect.

### Anticancer effects of CSEO, CLEO, CAEO, and limonene

**Fig. 4:**
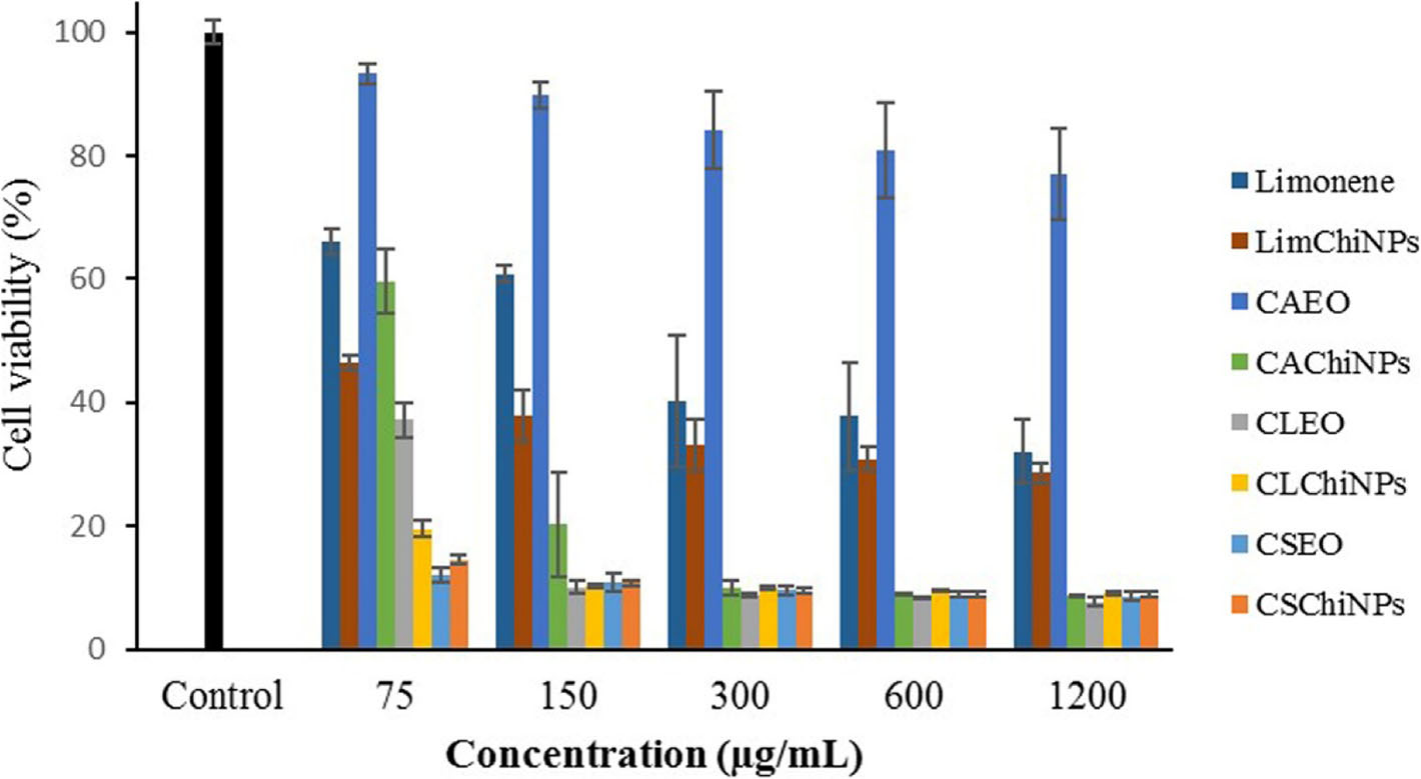
Anticancer effect of limonene and EOs of *C. aurantium*, *C. limon*, and *C. sinensis* (CAEO, CLEO, and CSEO) and chitosan nanoparticles contained them (LimChiNPs, CAChiNPs, CLChiNPs, and CSChiNPs) against A-375 cells. Data are presented as mean ± standard deviation (*n* = 3).

Figure 4 illustrates the cytotoxicity effects of limonene, CAEO, CLEO, and CSEO and their nanoformulated forms on A-375 cells; obtained IC_50_s are listed in Table 2. Interestingly, the viability of A-375 cells treated with CLChiNPs, CSEO, and CSChiNPs at all examined concentrations (150, 300, 600, and 1200 μg/mL) was reduced < 20%. IC_50_s of CLChiNPs, CSEO, and CSChiNPs were 0.124, 0.02, and 0.03 μg/mL. Besides, IC_50_s of LimChiNPs and CAChiNPs were observed at 30.24 and 55.00; there was no significant difference together (*p* > 0.05). However, their potency was significantly more than their no-formulated forms, i.e., limonene and CAEO, with IC_50_s of 246.05 and 10,564.00 μg/mL.

**Table 2:** Anticancer effects of samples against A-375 and MDA-MB-468

Samples	A-375			MDA-MB-468		
	LCL^*^	IC_50_^*^	UCL^*^	LCL	IC_**50**_	UCL
**limonene**	176.18	246.05	343.62	1636.44	2118.94	2743.71
**LimChiNPs**^**a**^	13.53	30.24	67.58	498.29	650.70	849.72
**CAEO**^**b**^	4832.01	10,564.00	23,095.00	781.41	2037.53	5312.86
**CAChiNPs**^**c**^	15.66	55.00	193.16	144.20	240.44	400.92
**CLEO**^**d**^	0.49	10.54	228.46	57.41	137.03	327.06
**CLChiNPs**^**e**^	3.270	0.124	0.05	10.41	40.32	156.14
**CSEO**^**f**^	0.01	0.02	0.03	139.97	168.00	201.65
**CSChiNPs**^**g**^	0.02	0.03	0.05	2.74	23.65	204.43

^a^ chitosan nanoparticles containing limonene, ^b^ *C. aurantium* EO, ^c^ chitosan nanoparticles containing *C. aurantium* EO, ^d^ *C. limon* EO, ^e^ chitosan nanoparticles containing *C. limon* EO, ^f^ *C. sinensis* EO, chitosan nanoparticles *C. sinensis* EO

* The half-maximal inhibitory concentration or IC_50_ (μg/mL) with lower and upper confidence limits; LCL and UCL

**Fig. 5:**
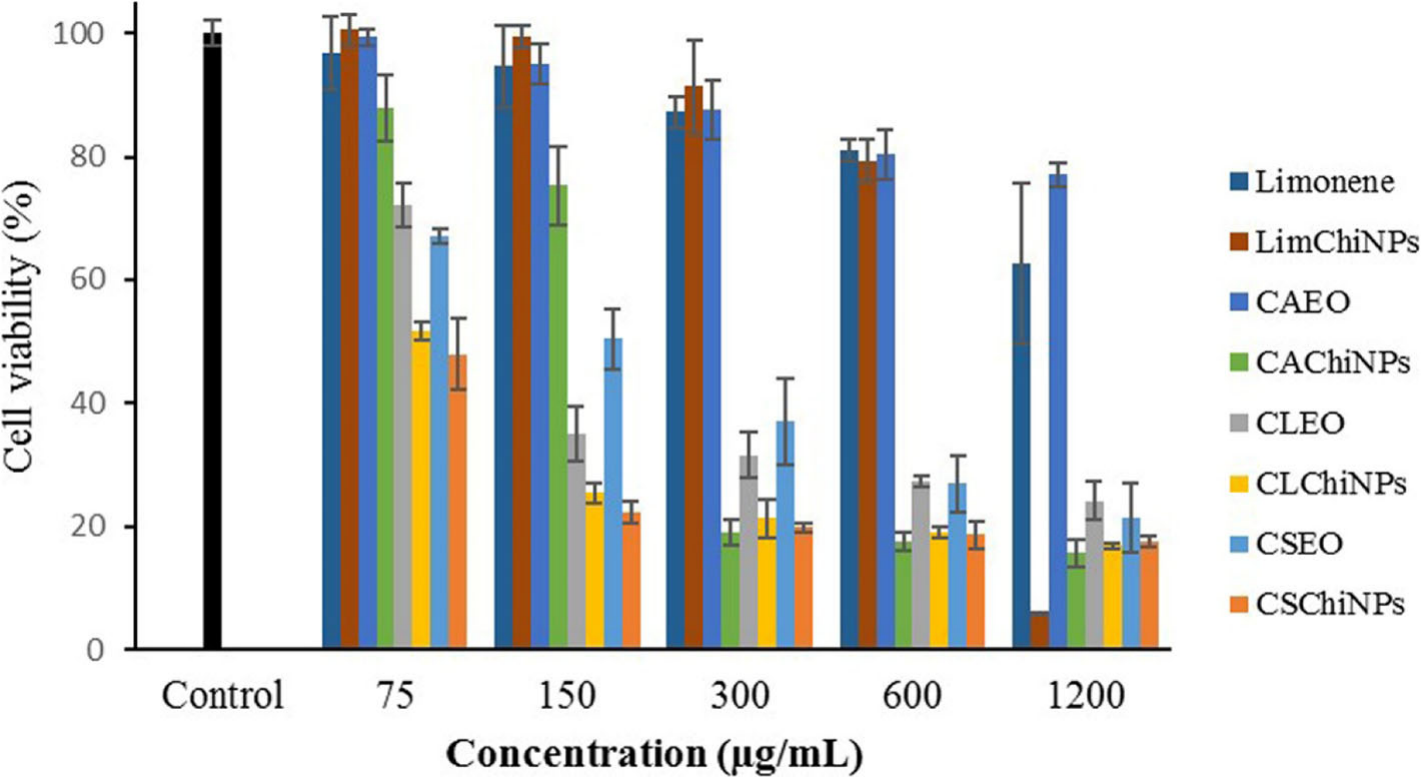
Anticancer effect of limonene and EOs of *C. aurantium*, *C. limon*, and *C. sinensis* (CAEO, CLEO, and CSEO) and chitosan nanoparticles contained them (LimChiNPs, CAChiNPs, CLChiNPs, and CSChiNPs) against MDA-MB-468. Data are presented as mean ± standard deviation (*n* = 3)

From Fig. 5, the cytotoxicity effects of limonene, CAEO, CLEO, and CSEO on MDA-MB-468 cells are depicted. Generally, the samples had less potency on the growth of this cell line than A-375 cells; viability of cells reduced to lower than 10% after treating with only LimChiNPs at a concentration of 1200 μg/mL. The best-obtained IC_50_s were related to CSChiNPs (23.65 μg/mL) and CLChiNPs (40.32 μg/mL). Moreover, IC_50_s of LimChiNPs and CAChiNPs (650.70 and 240.44 μg/mL) were significantly more potent than their non-formulated forms (2118.94 and 2037.53 μg/mL).

## Discussions

Obtained IC_50_ for CSEO (0.02 μg/mL) against A-375 in the current study was more potent than that reported in the literature, e.g., IC_50_s of *Ajuga chamaepity*, *Sideritis montana*, and *Eryngium campestre* EOs were reported at 67.44, 34.89, and 1.57 [28–30]. Besides, the efficacy of the most potent non-formulated sample against MDA-MB-468 in the current study (i.e., CLEO 137 μg/mL) was assessed as moderate compared to those published in the literature.; *Kelussia odoratissima* EO 85.00 μg/mL, *Zataria multiflora* EO 302 μg/mL, and *Mentha piperita* EO 2536 μg/mL [25, 31, 32].

Interestingly, no report was found on preparing chitosan nanoparticles containing CAEO, CLEO, CSEO, and limonene as anticancer agents on these cell lines in the literature. However, some reports on the preparation of some formulations have been found. For instance, chitosan microcapsules containing limonene with a particle size of 2–12 μm were reported [33]. Electrosprayed *Alyssum homolocarpum* seed gum nanoparticles containing limonene with a mean diameter of 65.68 ± 8.80 nm was reported in another research [34]. In a study, CAChiNPs with a particle size of 40 nm was proposed as mushroom food packaging; it significantly decelerated the rate of color change, weight loss, and firmness compared to fumigation with EO [35]. In another study, by incorporating CAEO in ChiNPs (20–60 nm), antioxidant and antimicrobial effects were improved [36]. Chitosan nanoemulsion containing CSEO as a green fruit juice preservative was also reported [37]. In another report, CLEO was encapsulated in chitosan/hicap with a mean particle size of 339.3 nm; it was concluded that due to the desirable physicochemical properties and thermal stability, this formulation could be used in medicine and food industries [38].

Furthermore, the preparation of nanoformulations to improve the anticancer effects of EOs has recently received much attention [39]. For instance, a study confirmed that by loading limonene into ChiNPs with a particle size of 339.5 nm, its antioxidant became more potent by improving its solubility [40]. CAEO nanoemulsion showed cytotoxicity against A549 cells with the IC_50_ value of 152 μg/mL [41]. Besides, isolated nanovesicles from *C. lemon* have been shown an inhibitory effect in a time-dependent manner on cancer cell proliferation in different tumor cell lines by inducing apoptotic cell processes [42]. It has also been proposed that loading limonene into solid lipid nanoparticles (one of the pharmaceutical drug delivery systems) can reduce growth percentages in cancer cells with a low toxic effect on the non-tumoral cell line [43]. In vitro cytotoxicity assay showed that D-limonene-loaded niosome had a noticeable anticancer effect than D-limonene against HepG2, MCF-7, and A549 cell lines [39].

In the current study, by preparing ChiNPs containing EOs/limonene, their anticancer effects were improved, e.g., IC_50_s of limonene against A-375 and MDA-MB-468 were obtained at 246 and 2118 μg/mL, while IC_50_s of its nanoformulated state (LimChiNPs) were observed at 30 and 650 μg/mL. CAEO did not show proper efficacy on both mentioned cell lines (IC_50_ 10,564 and 2037 μg/mL). However, after the preparation of ChiNPs containing CAEO, its IC_50_s were reduced dramatically to 55 and 240 μg/mL. Moreover, only IC_50_ of CSEO (0.02 μg/mL) against A-375 cells was not improved after preparation of its nanoformulation (CSChiNPs: 0.03 μg/mL); further investigation is needed at low concentrations. However, as the hydrophobic nature of CSEO, for its practical administration in vivo or clinical trials, it should be formulated; thus, its nanoformulated form (CSChiNPs) was the best sample against A-375 cells. Interestingly, IC_50_s of CSChiNPs and CLChiNPs (0.03 and 0.124 μg/mL) against A-375 were more potent than cisplatin with IC_50_ of 0.40–0.45 μg/mL [29, 44]. Their efficacies against MDA-MB-468 (23.65 and 40.32 μg/mL) were also comparable or more potent than cisplatin (IC_50_ 32.50 μg/mL) [45].

## Conclusions

Compounds of three EOs from the *Citrus* family, including *C. aurantium, C. limon,* and *C. sinensis,* were first identified using GC-MS analysis. Their anticancer effects were then investigated on human breast cancer and melanoma cell lines. After that, chitosan nanoparticles containing EOs and limonene (as their major ingredients) were prepared for improving their efficacies. Chitosan nanoparticles containing *Citrus sinensis* and *Citrus limon* essential oils with IC_50_s of 0.03 and 0.124 μg/mL on A-375 cells, and 23.65 and 40.32 μg/mL on MDA-MB-468 showed more potency than other samples. Thus two mentioned formulations could be considered as green anticancer agents in future studies.

## Data Availability

All data generated or analyzed during this study are included in this published article.

## Abbreviations

*GC–MS*: *Chromatography-Mass Spectrometry*
ATR-FTIR: Attenuated Total Reflection-Fourier Transform InfraRed
EO: Essential Oil
CAEO: *Citrus aurantium* essential oil
CLEO: *Citrus limon* essential oil
CSEO: *Citrus sinensis* essential oil
ChiNPs: Chitosan nanoparticles
CAChiNPs: Chitosan nanoparticles containing *Citrus aurantium* essential oil
CLChiNPs: Chitosan nanoparticles containing *Citrus limon* essential oil
CSChiNPs: Chitosan nanoparticles containing *Citrus sinensis* essential oil
